# Analysis of Changes in Glucose and Lipid Metabolism in Patients with Clinically Severe Obesity and Type 2 Diabetes Mellitus Undergoing Laparoscopic Sleeve Gastrectomy—Prospective Observational Study

**DOI:** 10.1007/s11695-023-06991-8

**Published:** 2023-12-18

**Authors:** Michał Wysocki, Magdalena Mizera, Izabela Karpińska, Kuba Ptaszkiewicz, Piotr Małczak, Magdalena Pisarska-Adamczyk, Michał Kania, Piotr Major

**Affiliations:** 1 Department of General Surgery and Surgical Oncology, Ludwik Rydygier Memorial Hospital in Cracow, Os. Zlotej Jesieni 1, 31-826 Cracow, Poland; 2 2nd Department of General Surgery, Jagiellonian University Medical College, Cracow, Poland; 3 Department of Medical Education, Jagiellonian University Medical College, Cracow, Poland; 4 Department of Metabolic Diseases and Diabetology, Jagiellonian University Medical College, Cracow, Poland

**Keywords:** Laparoscopic sleeve gastrectomy, Morbid obesity, Diabetes mellitus, Continuous glucose monitoring, Metabolic profile

## Abstract

**Introduction:**

We still lack studies providing analysis of changes in glucose and lipid metabolism after laparoscopic sleeve gastrectomy (LSG) in patients with type 2 diabetes mellitus (DM2). We aimed to investigate postoperative changes in glucose and lipid metabolism after LSG in patients with DM2.

**Material and Methods:**

Prospective, observational study included patients with BMI ≥ 35 kg/m^2^ and ≤ 50 kg/m^2^, DM2 < 10 years of duration, who were qualified for LSG. Perioperative 14-day continuous glucose monitoring (CGM) began after preoperative clinical assessment and OGTT, then reassessment 1 and 12 months after LSG. Thirty-three patients in mean age of 45 ± 10 years were included in study (23 females).

**Results:**

EBMIL before LSG was 17 ± 11.7%, after 1 month—36.3 ± 12.8%, while after 12 months—66.1 ± 21.7%. Fifty-two percent of the patients had DM2 remission after 12 months. None required then insulin therapy. 16/33 patients initially on oral antidiabetics still required them after 12 months. Significant decrease in HbA_1C_ was observed: 5.96 ± 0.73%; 5.71 ± 0.80; 5.54 ± 0.52%. Same with HOMA-IR: 5.34 ± 2.84; 4.62 ± 3.78; 3.20 ± 1.99. In OGTT, lower increase in blood glucose with lesser insulin concentrations needed to recover glucose homeostasis was observed during follow-ups. Overtime perioperative average glucose concentration in CGM of 5.03 ± 1.09 mmol/L significantly differed after 12 months, 4.60 ± 0.53 (*p* = 0.042). Significantly higher percentage of glucose concentrations above targeted compartment (3.9–6.7 mmol/L) was observed in perioperative period (7% ± 4%), than in follow-up (4 ± 6% and 2 ± 1%). HDL significantly rose, while triglyceride levels significantly decreased.

**Conclusions:**

Significant improvement in glucose and lipid metabolism was observed 12 months after LSG and changes began 1 month after procedure.

**Graphical Abstract:**

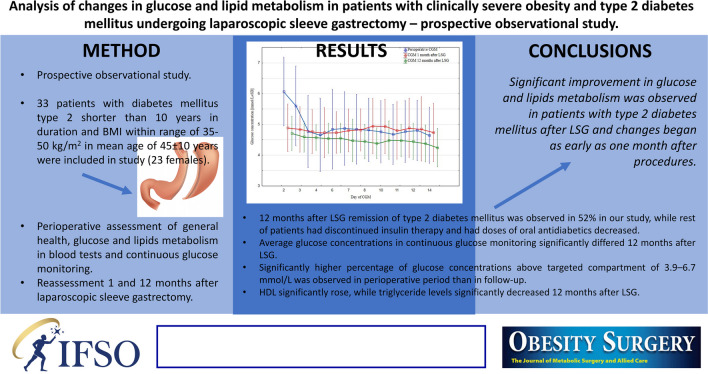

## Introduction

Obesity is the main civilization disease of the twenty-first century, contributing to the development of diabetes mellitus type 2 (DM2), to the deterioration of quality of life and to the shortening life expectancy [1–6]. Surgical treatment of morbid obesity is the only method giving lasting effects in terms of weight loss (bariatric effect) and a curative effect on DM2 (metabolic effect). DM2 resolves in 40–95% of patients, depending on its duration, severity of obesity, and the type of surgical procedure [7]. Observational studies and randomized control trials demonstrated that bariatric surgery performed with the intention to treat DM2 significantly improves glucose metabolism leading to remission of prediabetes and DM2 in a short-term follow-up and in the long-term controls [4, 8–21]. An improvement in control or remission of the obesity-related comorbidities is observed after all types of the bariatric surgeries. Most randomized trials among the patients with DM2, comparing the effectiveness of LSG and laparoscopic gastric bypass (two most commonly performed bariatric procedures), reported similar remission rates at various follow-ups after surgery, using variable criteria to define remission of DM2. Three RCTs recruited only patients with DM2 [22, 23], while others included a proportion of patients with DM2 at randomization [24–26]. Still, none of them comprises a comprehensive analysis of changes in metabolic profile. That is why we designed the study to investigate the immediate changes in glucose and lipids homeostasis, as well as 1 and 12 months after LSG, not only in one fasting blood glucose test, but in continuous glucose monitoring (CGM) and oral glucose tolerance test (OGTT). Examining daily trends in glucose levels leads to practical conclusion. Even in the newest recommendations, we could not find recommendations for antidiabetic treatment in the early postoperative period.

## Material and Methods

A prospective, observational study was designed and conducted between 2020 and 2023 in a tertiary referral, university bariatric center. Study included the patients with morbid obesity and DM2, qualified to LSG, between 18 and 65 years of age, with body mass index (BMI) on the day of surgery ≥ 35 kg/m^2^ and ≤ 50 kg/m^2^. Patients on oral antidiabetic drugs or insulin therapy with DM2 duration shorter than 10 years were included in the study. All patients signed informed consent to participate in the study. Patients were excluded, when diagnosed with diabetes mellitus other than type 2; qualified to revisional bariatric procedures (which means patients who had previously underwent other type of bariatric surgery); diagnosed with psychiatric illness or intellectual disability or alcohol, drugs, or other psychoactive substances abuse; and had history of steroid treatment, endocrine disorders related to impaired glucose metabolism, inflammatory bowel diseases, malabsorption syndrome, liver cirrhosis (Child B or C), chronic kidney disease, chronic viral infections (e.g., human immunodeficiency virus, hepatitis B or C), autoimmune diseases or history of cancer treatment within the past 5 years.

The study was conducted in three phases:

- 1 — Preparations and preoperative clinical assessment — included full clinical examination, body weight, BMI, waist and hips circumferences measurement, baseline evaluation of biochemical and hormonal parameters (glycated hemoglobin — HbA_1c_, OGTT, insulin, C-peptide, lipids, nutritional parameters).
- 2 — Perioperative CGM — continuous monitoring of interstitial glucose concentration in patients’ subcutaneous tissue, beginning 1 day prior to LSG up to the 13th postoperative day.
- 3 — Follow-up 1 month and 12 months after LSG — clinical reassessment, including full clinical examination, body weight, BMI, waist and hips circumferences measurement, evaluation of selected biochemical parameters (HbA_1c_, OGTT, insulin, C-peptide, lipids, nutritional parameters), CGM.

### Primary Outcome

Comparison of changes in glucose metabolism and daily glycemic fluctuations in CGM in patients with clinically severe obesity and DM2 perioperatively, 1 and 12 months after LSG.

### Secondary Outcome

Changes in lipid profile in with clinically severe obesity and DM2 perioperatively, 1 and 12 months after LSG.

DM2 remission was defined as no need for antidiabetic therapy for 3 months with fasting glucose within normal range and HbA_1c_ level is < 6.5%, accordingly with Consensus Report: Definition and Interpretation of Remission in Type 2 Diabetes by Riddle et al. [27].

Remission of dyslipidemia was defined as low-density lipoprotein (LDL) cholesterol < 3.0 mmol/L, triglycerides < 1.7 mmol/L, and high-density lipoprotein (HDL) cholesterol > 1.2 mmol/L (women) or > 1.0 mmol/L (men) and no need for medications [28].

In the 6-month preoperative period, patients were referred to clinical dietician and psychologist for evaluation and preparation for operation. Patients were not put on restrictive hypocaloric diet, but advised about frequent meals of smaller volume and underwent healthy eating training during at least two sessions with clinical dietician.

Continuous glucose monitoring was done using Freestyle Libre (Abbott, USA). Sensors were applied on anterolateral aspect of patients’ arm. Sensor’s needle was place in subcutaneous tissue and either patient or medical professionals were gathering data from sensors with paired devices. Targeted compartment for interstitial glucose concentrations was 3.9–6.7 mmol/L (70–120 mg%). Mean daily glucose concentration on the first day of CGM was considered unreliable, because CGM was implemented after OGTT on the same day and CGM device was also calibrating on the first day of use. Sensors were applied 1 day prior to LSG and continued for a total of 14 days. Postoperatively, 1 and 12 months after sensors were applied after completed OGTT. Device’s software allowed for analysis of the following variables:

- Average glucose concentration overtime — i.e., mean concentration of all measurements
- Percentage of glucose concentration measurements above targeted compartment (> 6.7 mmol/L; > 120 mg%) during the whole duration of CGM
- Percentage of glucose concentration measurements in targeted compartment (3.9–6.7 mmol/L; 70–120 mg%) during the whole duration of CGM
- Percentage of glucose concentration measurements below targeted compartment (< 3.9 mmol/L; < 70 mg%) during the whole duration of CGM
- Count of low glucose concentration events, i.e., the number of events of glucose concentration < 3.9 mmol/L
- Time of low glucose event, i.e., duration of glucose concentration < 3.9 mmol/L; < 70 mg%
- Daily glucose concentration — mean of all interstitial glucose concentrations on the particular day of CGM

Homeostatic model assessment for insulin resistance index (HOMA-IR) was calculated using the formula: fasting glucose (mmol/L) * fasting insulin (mU/L)/22.5 [29, 30].

Percent of excess body mass index loss (EBMIL) derived from the difference of body mass indexes divided by the initial BMI minus 25 and expressed as percentage. Percent of weight loss (%WL) was calculated by dividing the absolute kilograms lost by the patient’s initial weight.

### Statistics

Statistical analysis was performed using Tibco STATISTICA 13.3. Qualitative data were compared using chi-square tests. Quantitative data were presented as means with standard deviations (SD). Comparative analysis was done with repeated measurements ANOVA with post hoc tests. *p*-value of less than 0.05 was considered statistically significant.

### Ethics

All procedures have been performed in accordance with the ethical standards laid down in the 1964 Declaration of Helsinki and its later amendments. Informed consent for surgical treatment was obtained from all patients before surgery. The study was approved by Jagiellonian University Bioethics Committee (approval number 1072.6120.300.2019).

### Patients

Thirty-three patients were included in the study, 23 females, 10 males. Patients’ mean age was 45 ± 10 years. General characteristics of study population are presented in Table 1.

**Table 1:** General characteristics

Mean maximal BMI, kg/m^2^ ± SD		47.5 ± 5.9
Mean maximal weight, kg ± SD		132.9 ± 21.7
Mean preoperative BMI, kg/m^2^ ± SD		43.4 ± 4.4
Mean preoperative weight, kg ± SD		121.3 ± 15.9
ASA, *n* (%)	2	4 (12%)
	3	29 (88%)
Median duration of DM2, years (Q1–Q3)		3 (1–5)
Dyslipidemia, *n* (%)		15 (45%)
Non-alcoholic fatty liver disease, *n* (%)		19 (58%)
Hypertension, *n* (%)		28 (85%)
Cardiovascular disease, *n* (%)		3 (9%)
Obstructive sleep apnea, *n* (%)		8 (24%)
Degenerative joint disease, *n* (%)		16 (48%)
Tobacco smoking, *n* (%)		7 (21%)

*BMI* body mass index

*DM2* type 2 diabetes mellitus

*ASA* grade in America Society of Anesthesiologists scale

## Results

### Bariatric Results

**Fig. 1:**
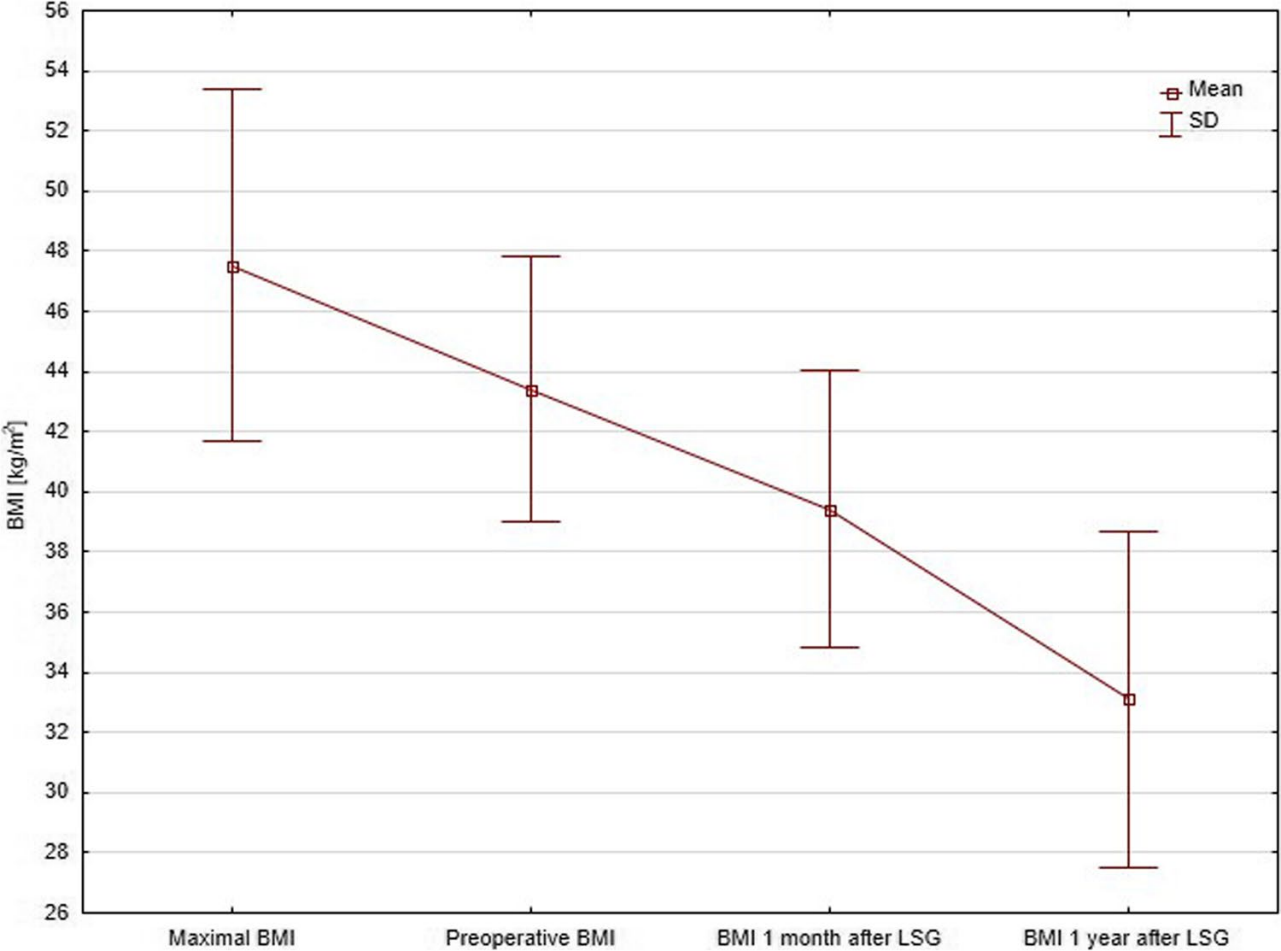
Changes in patients’ body mass indexes during study period

All patients underwent LSG. None of patients developed serious postoperative morbidity, but two patients had persistent symptoms of gastroesophageal reflux in the postoperative period. All patients included in the study completed 1- and 12-month postoperative follow-up. BMI gradually decreased during the study period, as depicted in Fig. 1. Changes in mean patients’ weight, BMI, and EBMIL are presented in Table 2. In repeated measurements ANOVA, all indexes differed significantly with *p*-value of Bonferroni’s post hoc test < 0.001.

**Table 2:** Changes in registered measurements and laboratory tests

	Preoperative	After 1 month	After 12 months	*p*-value
Anthropometric measurements				
Mean BMI, kg/m^2^ ± SD	43.4 ± 4.4	39.4 ± 4.6	33.1 ± 5.6	**1 vs. 2 < 0.001** **2 vs. 3 < 0.001** **1 vs. 3 < 0.001**
Mean weight, kg ± SD	121.3 ± 15.9	110.2 ± 15.6	94.1 ± 20.0	**1 vs. 2 < 0.001** **2 vs. 3 < 0.001** **1 vs. 3 < 0.001**
Mean EBMIL, % ± SD	17.0 ± 11.7	36.3 ± 12.8	66.1 ± 21.7	**1 vs. 2 < 0.001** **2 vs. 3 < 0.001** **1 vs. 3 < 0.001**
Mean WL, % ± SD	8.2 ± 6.4	16.8 ± 6.0	30.2 ± 8.7	**1 vs. 2 < 0.001** **2 vs. 3 < 0.001** **1 vs. 3 < 0.001**
Mean waist circumference, cm ± SD	127.6 ± 11.3	123.5 ± 10.8	105.7 ± 15.7	1 vs. 2 0.318 **2 vs. 3 0.035** **1 vs. 3 < 0.001**
Mean hips circumference, cm ± SD	132.8 ± 9.2	127.0 ± 9.4	115.4 ± 9.2	**1 vs. 2 0.013** **2 vs. 3 < 0.001** **1 vs. 3 < 0.001**
Glucose metabolism				
Diabetes remission, *n* (%)	0	0	17 (52%)	n/a
Insulin therapy, *n* (%)	5 (15%)	4 (12%)	0	n/a
Oral antidiabetics, *n* (%)	33 (100%)	17 (52%)	16 (48%)	n/a
GLP-1 analogs, *n* (%)	4 (12%)	3 (9%)	2 (6%)	0.693
HbA_1C_, % ± SD	5.96 ± 0.73	5.71 ± 0.80	5.54 ± 0.52	**1 vs. 2 0.022** **2 vs. 3 < 0.001** **1 vs. 3 < 0.001**
Mean fasting blood glucose, mmol/L ± SD	6.75 ± 3.89	5.54 ± 1.08	5.08 ± 0.92	**1 vs. 2 0.038** **2 vs. 3 0.047** **1 vs. 3 0.013**
Mean fasting insulin, mU/L ± SD	24.25 ± 15.38	17.24 ± 12.26	14.06 ± 8.76	**1 vs. 2 0.004** 2 vs. 3 0.908 **1 vs. 3 0.001**
Mean fasting C-peptide, ng/mL ± SD	10.76 ± 39.26	3.70 ± 1.18	3.05 ± 1.20	**1 vs. 2 < 0.001** 2 vs. 3 0.095 **1 vs. 3 < 0.001**
Mean HOMA-IR, ± SD	5.34 ± 2.84	4.62 ± 3.78	3.20 ± 1.99	**1 vs. 2 0.001** 2 vs. 3 0.568 **1 vs. 3 < 0.001**
Mean OGTT — blood glucose after 1 h, mmol/L ± SD	11.60 ± 5.58	11.10 ± 3.00	9.34 ± 3.35	1 vs. 2 0.406 2 vs. 3 0.211 **1 vs. 3 0.011**
Mean OGTT — insulin after 1 h, mU/L ± SD	122.91 ± 94.05	138.71 ± 11.77	92.97 ± 58.71	1 vs. 2 0.380 **2 vs. 3 0.002** **1 vs. 3 0.044**
Mean OGTT — C-peptide after 1 h, ng/mL ± SD	11.12 ± 5.02	19.66 ± 17.20	12.22 ± 4.86	**1 vs. 2 < 0.001** **2 vs. 3 < 0.001** 1 vs. 3 0.451
Mean OGTT — blood glucose after 2 h, mmol/L ± SD	7.76 ± 2.67	8.08 ± 1.37	6.00 ± 3.48	**1 vs. 2 < 0.001** 2 vs. 3 0.382 **1 vs. 3 0.008**
Mean OGTT — insulin after 2 h, mU/L ± SD	68.67 ± 45.78	21.44 ± 17.80	33.35 ± 10.71	**1 vs. 2 < 0.001** 2 vs. 3 0.287 **1 vs. 3 0.037**
Mean OGTT — C-peptide after 2 h, ng/mL ± SD	10.33 ± 3.03	6.88 ± 3.43	6.07 ± 3.31	**1 vs. 2 0.001** 2 vs. 3 0.440 **1 vs. 3 < 0.001**

*BMI* body mass index

*EBMIL* excess body mass index loss

*SD* standard deviation

*HbA*_*1C*_ glycated hemoglobin level

*HOMA-IR* homeostatic model assessment for insulin resistance index

*OGTT* oral glucose tolerance test with 75 g of glucose taken orally

*p*-values that are statistically significant (i.e. <0.05) were bolded

### Primary Outcomes

All registered measurements and repeated laboratory tests are presented in Table 2. After 12 months, complete DM2 remission was observed in 17 patients (52%). None of patients 12 months after LSG required insulin therapy. Out of 33 patients on oral antidiabetics, 16 patients still required them after 12 months, but doses significantly dropped. Out of 4 patients on GLP-1 analogs, 2 still needed them after 12 months. HbA_1C_ perioperatively was 5.96 ± 0.73, then significantly decreased as follows: 1 month after LSG was 5.71 ± 0.80 and 12 months after LSG — 5.54 ± 0.52. Significant decrease in mean HOMA-IR was observed between preoperative (5.34 ± 2.84), 1 month (4.62 ± 3.78), and 12 months after LSG indexes (3.20 ± 1.99). Improvement in glucose homeostasis is reflected in results of 2-h OGTT with 75 g of glucose taken orally. Better metabolic response was detected, pictured by lower increase in blood glucose after 1 and 2 h during study phases as presented in Table 2 with lesser insulin concentrations needed to recover glucose homeostasis after OGTT.

**Fig. 2:**
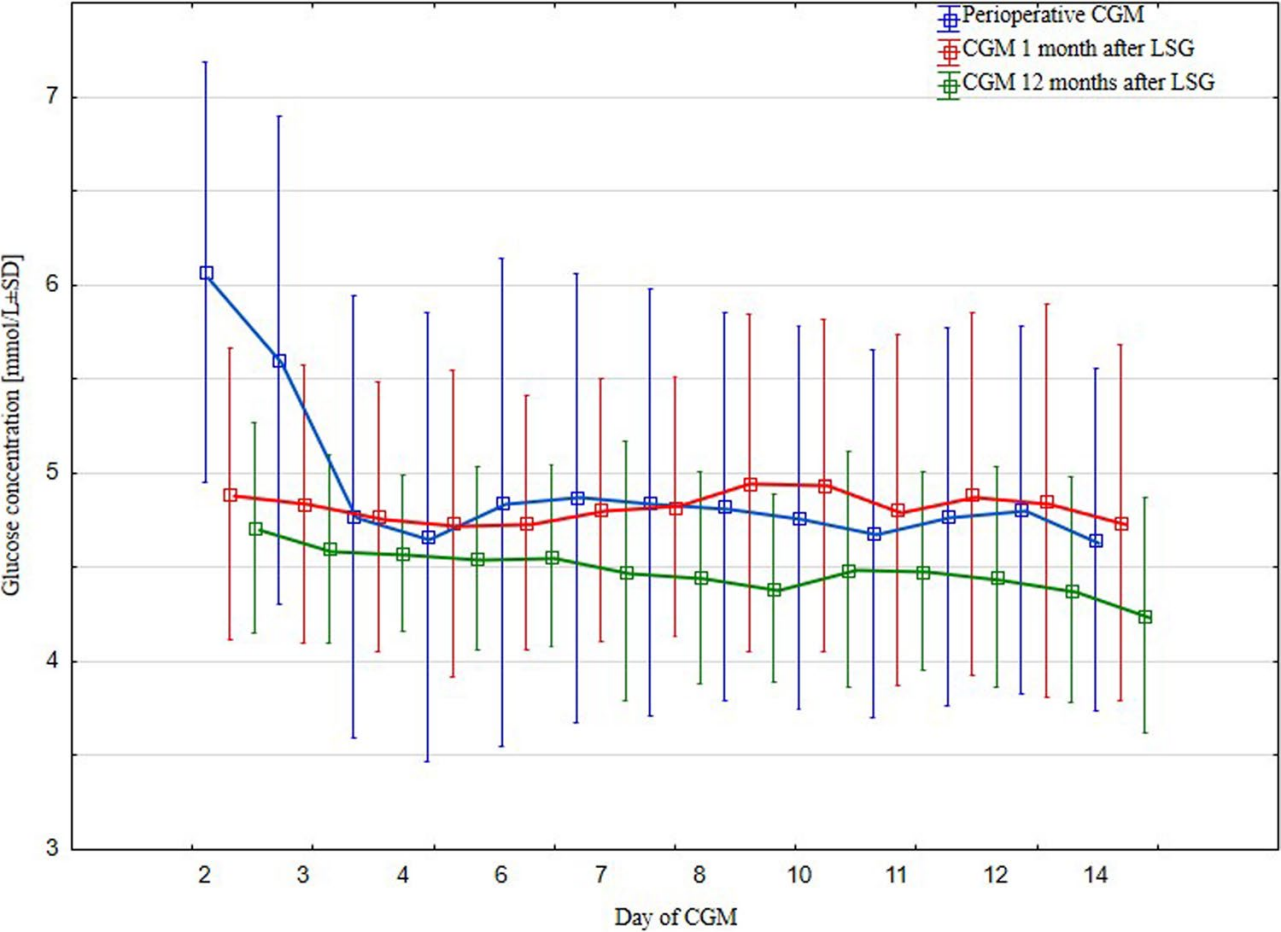
Mean daily glucose concentrations (mmol/L ± SD) during study phases

Table 3 comprises results of continuous glucose monitoring during follow-ups. Average glucose concentration overtime in perioperative CGM was 5.03 ± 1.09 mmol/L and significantly differed from average concentration of 4.60 ± 0.53 after 12 months (*p* = 0.042). Average concentration after 1 month did not differ from other CGMs. Significantly higher percentage of glucose concentrations above targeted compartment (3.9–6.7 mmol/L; 70–120 mg%) was observed in perioperative period (7 ± 4%), than in 1 month after LSG (4 ± 6%), and after 12 months (2 ± 1%). Percentage of glucose concentration measurements in a targeted compartment in the perioperative period was 69 ± 23%, 1 month — 77 ± 17%, and 12 months after LSG — 72 ± 17%. Percentage of glucose concentrations below targeted compartment in the perioperative period was 25 ± 23%, 1 month — 19 ± 2%, and 12 months after LSG — 26 ± 17%. Regretfully, differences in mean percentages of glucose concentration measurements regarding targeted compartment did not reach statistical significance. Mean count of low glucose concentration events at study phases was as follows: 14 ± 12, 16 ± 12, and 20 ± 11. Average time of low glucose events in study phases is presented in Table 3. In the next days, i.e., on the second and the third day of CGM, the surgical trauma was reflected in significantly higher mean daily glucose concentrations than in follow-ups. On the fourth day of CGM, patients were routinely alimented on semi-liquid diabetic diet, then continued after hospital discharge. On the fourth day of perioperative CGM, patients were returning to oral antidiabetics and insulin therapy. Average daily glucose concentrations in the next days of perioperative CGM were comparable to those measured after 1 month. Average daily glucose concentrations after 12 months seemed to be lower than in perioperative CGM and in CGM 1 month after LSG, but regretfully in post hoc tests of repeated measurements ANOVA those differences did not reach statistical significance. Excursions of mean daily glucose concentrations during study CGMs are presented in Fig. 2.

**Table 3:** Results of continuous glucose monitoring during three study phases

	Perioperative	After 1 month	After 12 months	*p*-value
Average glucose concentration overtime, mmol/L ± SD	5.03 ± 1.09	4.86 ± 0.78	4.60 ± 0.53	1 vs. 2 0.271 2 vs. 3 0.287 **1 vs. 3 0.042**
Mean percentage of glucose concentration measurements above targeted compartment (3.9–6.7 mmol/L; 70–120 mg%) ± SD	7 ± 4	4 ± 6	2 ± 1	**1 vs. 2 0.007** 2 vs. 3 0.491 **1 vs. 3 < 0.001**
Mean percentage of glucose concentration measurements in targeted compartment (3.9–6.7 mmol/L; 70–120 mg%) ± SD	69 ± 23	77 ± 17	72 ± 17	1 vs. 2 0.297 2 vs. 3 0.366 1 vs. 3 0.989
Mean percentage of glucose concentration measurements below targeted compartment (3.9–6.7 mmol/L; 70–120 mg%) ± SD	25 ± 23	19 ± 2	26 ± 17	1 vs. 2 0.563 2 vs. 3 0.323 1 vs. 3 0.906
Mean count of low glucose concentration events, *n* ± SD	14 ± 12	16 ± 12	20 ± 11	1 vs. 2 0.849 2 vs. 3 0.376 1 vs. 3 0.151
Average time of low glucose event, min ± SD	194 ± 142	181 ± 103	228 ± 106	1 vs. 2 0.574 2 vs. 3 0.084 1 vs. 3 0.237
Mean daily glucose concentration on day 1, mmol/L ± SD	6.04 ± 1.17	5.62 ± 0.97	5.37 ± 0.76	n/a
Mean daily glucose concentration on day 2, mmol/L ± SD	6.07 ± 1.12	4.89 ± 0.78	4.71 ± 0.56	**1 vs. 2 < 0.001** 2 vs. 3 0.991 **1 vs. 3 < 0.001**
Mean daily glucose concentration on day 3, mmol/L ± SD	5.60 ± 1.30	4.84 ± 0.74	4.60 ± 0.50	**1 vs. 2 < 0.001** 2 vs. 3 0.859 **1 vs. 3 < 0.001**
Mean daily glucose concentration on day 4, mmol/L ± SD	4.77 ± 1.18	4.77 ± 0.72	4.57 ± 0.41	1 vs. 2 0.826 2 vs. 3 0.818 1 vs. 3 0.999
Mean daily glucose concentration on day 5, mmol/L ± SD	4.66 ± 1.19	4.74 ± 0.81	4.55 ± 0.48	1 vs. 2 0.377 2 vs. 3 0.826 1 vs. 3 0.733
Mean daily glucose concentration on day 6, mmol/L ± SD	4.85 ± 1.30	4.74 ± 0.68	4.56 ± 0.48	1 vs. 2 0.971 2 vs. 3 0.896 1 vs. 3 0.778
Mean daily glucose concentration on day 7, mmol/L ± SD	4.87 ± 1.20	4.81 ± 0.69	4.48 ± 0.69	1 vs. 2 0.875 2 vs. 3 0.182 1 vs. 3 0.401
Mean daily glucose concentration on day 8, mmol/L ± SD	4.85 ± 1.14	4.82 ± 0.69	4.45 ± 0.56	1 vs. 2 0.983 2 vs. 3 0.149 1 vs. 3 0.206
Mean daily glucose concentration on day 9, mmol/L ± SD	4.83 ± 1.03	4.95 ± 0.90	4.39 ± 0.50	1 vs. 2 0.822 **2 vs. 3 0.020** 1 vs. 3 0.084
Mean daily glucose concentration on day 10, mmol/L ± SD	4.76 ± 1.02	4.94 ± 0.88	4.49 ± 0.63	1 vs. 2 0.447 2 vs. 3 0.572 1 vs. 3 0.075
Mean daily glucose concentration on day 11, mmol/L ± SD	4.68 ± 0.98	4.81 ± 0.93	4.48 ± 0.52	1 vs. 2 0.903 2 vs. 3 0.609 1 vs. 3 0.859
Mean daily glucose concentration on day 12, mmol/L ± SD	4.77 ± 1.00	4.89 ± 0.96	4.45 ± 0.59	1 vs. 2 0.955 2 vs. 3 0.288 1 vs. 3 0.438
Mean daily glucose concentration on day 13, mmol/L ± SD	4.81 ± 0.98	4.85 ± 1.05	4.38 ± 0.60	1 vs. 2 0.888 2 vs. 3 0.228 1 vs. 3 0.093
Mean daily glucose concentration on day 14, mmol/L ± SD	4.65 ± 0.91	4.74 ± 0.95	4.14 ± 0.63	1 vs. 2 0.982 2 vs. 3 0.183 1 vs. 3 0.129

*p*-values that are statistically significant (i.e. <0.05) were bolded

### Secondary Outcomes

Results of repeated measurements of lipid panel and blood pressure are presented in Table 4. Remission of dyslipidemia was observed in 7 out of 15 patients (46.7%) 1 year after LSG. Despite that no significant changes were found in total cholesterol and LDL levels at study follow-ups, the level of HDL cholesterol had significantly risen. Preoperative level was 1.23 ± 0.28 mmol/L, 1 month after — 1.49 ± 1.90 mmol/L, and 12 months after — 1.51 ± 0.36 mmol/L. Preoperative triglyceride level was 1.72 ± 0.47 mmol/L, then significantly dropped to 1.37 ± 0.46 mmol/L after 12 months. All patients with hypertension were adequately treated in perioperative period, while 12 months after 12/28 (43%) patients did not require any antihypertensive treatment. Systolic blood pressure gradually dropped, but repeated measurements ANOVA revealed no significant differences.

**Table 4:** Changes in registered measurements and laboratory tests

	Preoperative	After 1 month	After 12 months	*p*-value
Lipid panel				
Remission of dyslipidemia, *n* (%)	n/a	0	7/15 (46.7%)	n/a
Mean total cholesterol, mmol/L ± SD	4.88 ± 1.00	4.75 ± 1.52	5.23 ± 0.83	1 vs. 2 0.052 **2 vs. 3 < 0.001** 1 vs. 3 0.279
Mean LDL, mmol/L ± SD	3.17 ± 0.98	6.80 ± 2.63	3.23 ± 0.85	1 vs. 2 0.140 2 vs. 3 0.114 1 vs. 3 0.999
Mean HDL, mmol/L ± SD	1.23 ± 0.28	1.49 ± 1.90	1.51 ± 0.36	**1 vs. 2 0.004** **2 vs. 3 < 0.001** **1 vs. 3 < 0.001**
Mean triglycerides, mmol/L ± SD	1.72 ± 0.47	1.62 ± 0.97	1.37 ± 0.46	**1 vs. 2 0.011** 2 vs. 3 < 0.999 **1 vs. 3 0.001**
Hypertension				
Remission of hypertension, *n* (%)	n/a	0	12/28 (43%)	n/a
Mean systolic blood pressure in hypertensive patients, mmHg ± SD	140 ± 14	129 ± 15	121 ± 16	1 vs. 2 0.165 2 vs. 3 0.618 1 vs. 3 0.618
Mean diastolic blood pressure in hypertensive patients, mmHg ± SD	84 ± 9	87 ± 7	82 ± 8	1 vs. 2 0.336 2 vs. 3 0.999 1 vs. 3 0.113
Mean systolic blood pressure in study population, mmHg ± SD	138 ± 14	127 ± 15	124 ± 18	1 vs. 2 0.216 2 vs. 3 0.999 1 vs. 3 0.999
Mean diastolic blood pressure in study population, mmHg ± SD	82 ± 8	87 ± 7	84 ± 9	1 vs. 2 0.336 2 vs. 3 0.999 1 vs. 3 0.113

*SD* standard deviation

*LDL* low-density lipoprotein cholesterol

*HDL* high-density lipoprotein cholesterol

*p*-values that are statistically significant (i.e. <0.05) were bolded

## Discussion

The presented study investigated changes in glucose homeostasis and lipid profile of morbidly obese patients with DM2 qualified to LSG in the perioperative period, 1 and 12 months after LSG. EBMIL after 12 months was 66.1 ± 21.7%. Twelve months after LSG remission of DM2 was observed in 52%. None of patients required then insulin therapy. Forty-eight percent of patients still required oral antidiabetics after 12 months, but doses significantly dropped. Significant decrease in HbA_1C_ and HOMA-IR was observed during study follow-ups. In the OGTT, lower increase in blood glucose after 1 and 2 h with lesser insulin concentrations needed to recover glucose homeostasis was observed during follow-ups. Overtime average glucose concentrations in CGM significantly decreased during follow-ups. On the second and third day of CGM, the surgical trauma was reflected in significantly higher mean daily glucose concentrations in perioperative period than in later follow-ups. Average daily glucose concentrations in the next days of perioperative CGM were comparable to those measured after 1 month. Average daily glucose concentrations after 12 months seemed to be lower than in perioperative CGM and in CGM 1 month after LSG, but regretfully those differences did not reach statistical significance. Lipid metabolism improved in terms of HDL and triglycerides.

Twelve months after LSG, the glucose metabolism significantly improves in diabetic and nondiabetic patients, with lower fasting glucose, insulin, c-peptide, HOMA-IR, and HbA_1c_. One-hundred and fifty patients that had undergone LSG were included in study by Gjessing et al., but only 17% of patients in their cohort had DM2 [31]. Nevertheless, overall insulin level 12 months after surgery dropped from 9.0 (6.1–13.7) to 3.3 (2.0–6.7) mU/L. HOMA-IR decreased from 2.3 (1.5–3.4) to 0.7 (0.5–1.4). HbA_1c_ improved from 5.7 (5.4–6.1%) to 5.2% (5.0–5.5%). DM2 resolved in 65% of diabetic patients [31]. Overall, DM2 resolves in 40–95% of patients after bariatric surgeries as mentioned, depending on its duration, severity of obesity, and the type of surgical procedure [7].

There have been a few CGM studies focusing on patients with DM2 after LSG. Capoccia and colleagues analyzed CGM from 20 patients with DM2 who showed complete diabetes remission after LSG [32]. Jimenez et al. presented CGM data from eight patients with diabetes who showed remission after LSG — those patients spent 0.4% of their monitored time above 10.0 mmol/L and 3.2% below 3.9 mmol/L [33]. In another study by Nosso et al., 11 patients with DM2 showing remission had glucose levels above 8.9 mmol/L for 10% of the CGM and below 3.3 mmol/L for 1% of the CGM [34]. LSG in studies cited above was compared with laparoscopic Roux-en-Y gastric bypass (LRYGB), and appeared to produce less glucose fluctuation than LRYGB and less postprandial hypoglycemia, but still hypoglycemic events were present during fasting period. Same was demonstrated by our study.

Hypoglycemia events, including severe hypoglycemia, are very common phenomena after LRYGB, one-anastomosis gastric bypass, and single-anastomosis duodenoileal bypass, but surprisingly occur also after LSG. We demonstrated it in our CGMs. Other published works had also raised this issue [35, 36]. However, most of the episodes after LSG are asymptomatic [35, 36]. Dumping syndrome is frequently described after bariatric surgery. It arises from the rapid emptying of undigested gastric contents into the small intestine. It can be classified into early and late dumping [37]. Early dumping occurs within 1 h after food intake and symptoms are hypotension, sometimes syncope with subsequent autonomic stress response, and also accompanied by gastrointestinal symptoms such as abdominal distention, painful cramps, nausea, and diarrhea. Late dumping is a phenomenon of our interest in the context of DM2. About 2 h after meal intake, after rapid hyperglycemia, there is a reactive hypoglycemia event, presenting with palpitations, sweating, tremor, irritability, or even unconsciousness. While early dumping often occurs in isolation, the solitary late dumping is a rare phenomenon [38]. Nielsen et al. investigated the prevalence of dumping up to 4.5 years after LRYGB in 1429 patients [39]. Early dumping was present in 9.4% of the patients, while reactive hypoglycemia, i.e., late dumping, in 6.6% of the cases and usually it was fully symptomatic. Of patients in their study, 3.4% had both early and late dumping. Incidence diminished with time after operations. In restrictive bariatric surgeries, such as LSG, the incidence rates are generally low. Tzovaras et al. assessed the incidence of dumping in 31 patients 6 weeks after sleeve gastrectomy, using the Sigstad score in combination with an OGTT [40]. About one-third presented symptoms of dumping. Papamargeritis et al. examined incidence of dumping 6 to 12 months after LSG in a group of 12 patients, using a 75-g OGTT combined questionnaires [41]. After 6 months, 24% had early symptoms, while after 12 months 25% of patients were diagnosed with late dumping. These figures for late dumping after LSG are remarkably higher than the incidence rates reported in large cohorts after LRYGB (as indicated above), but sample size of studies for LSG is definitely small. Several authors suggest that the reactive hypoglycemia occurs more frequently in patients with preoperative diabetes mellitus type 2 [42]. However, not all studies support this thesis. Emous et al. investigated 351 patients, 96 of them had preoperative type 2 diabetes [43]. No difference in its prevalence 2 to 3 years after RYGB was reported between patients without DM2, with postoperative normalized glycemia and with persistent DM2. As opposite, Padoin et al. found higher rates of hypoglycemia 1 year after RYGB in 49 patients with preoperative DM2 (44.9%) vs. 54 patients without preoperative DM2 (5.6%) [44]. The authors hypothesized that the higher incidence of hypoglycemia in the preoperative DM2 group might be related to the gastrointestinal neuropathy, but supporting data are lacking. Regretfully, we did not find data on the incidence and severity of hypoglycemic events in specific group of patients, that is, in patients with DM2 after LSG. None of our patients required rehospitalization or emergency department visit due to hypoglycemia; same none of the patients experienced any complication of OGTT. Nevertheless, patients required modification of antidiabetic medication as early as at the time of 1-month follow-up. Further studies are needed, preferable with use of CGM to determine the significance of those events in this specific population.

Long-term studies demonstrated a reduction in cardiovascular risk and mortality in diabetic patients undergoing bariatric surgery [45, 46]. We demonstrated decrease in HbA_1C_ and HOMA-IR. Lipid metabolism improved in terms of HDL and triglycerides after 12 months. The improvement in lipid profile is a result of multiple mechanisms, such as weight loss and improved insulin resistance, but also increased bile acids excretion and increased levels of GLP-1 [47, 48].

LSG since its introduction in 2001 became the most commonly performed bariatric procedure. Well, it offers sufficient results to morbidly obese patients with DM2. Yet, short observation of 22 years since its introduction still leaves doubts about long-lasting effects, especially about the metabolic efficacy. Implementation of CGM and OGTT assessment of glucose metabolism in patients with DM2 undergoing LSG can provide useful information about patients’ glucose metabolism status and can be a valuable tool in their postoperative management.

There is a lively discussion on the topic of the accuracy of CGM systems and differences of interstitial glucose and blood glucose measurements. The overall mean absolute relative difference (MARD) of our CGM device’s measurements relative to blood glucose measurements was 12.7 ± 9.3% in study by Nakagawa et al. [49], which is similar to a previously reported value of 11.1% relative to venous blood glucose [50]. Two hours postprandial MARD was 11.3 ± 13.4%, and similar to that preprandial values (12.5 ± 10.7%) [49]. The accuracy of CGM device used in our study was previously found not to be affected by BMI, age, type of diabetes, sensor insertion site, insulin administration, or HbA_1c_ level [51]. We hope that evaluation of glycemic variability in morbidly obese patients with DM2 was reliable, but some studies suggested that accuracy is diminishing when blood glucose is < 100 mg% giving MARD of 13–25.1% [49, 52].

### Limitations

The study is limited by several factors. We did not collect data concerning daily food intake, adherence to dietary and vitamin supplementation advisory, and physical activity, which could have influenced the results. Open-label design could interfere with patients dietary and lifestyle habits. The study has a relatively small sample size, and selection of patients with BMI 35–50 kg/m^2^ with DM2 lasting less than 10 years could cause selection bias, but rationale was to achieve comparability in impairment of glucose and lipid metabolism between individuals included in the research. Also, patients with severe DM2 were more likely qualified to LRYGB in bariatric center, where the study was conducted. That causes the bias to include less severe cases of DM2 undergoing LSG in the following study.

## Conclusions

Twelve months after LSG, remission of DM2 was observed in 52%, while the rest of patients had discontinued insulin therapy and had doses of oral antidiabetics decreased. Significant decrease in HbA_1C_ and HOMA-IR was observed 1 year after LSG, and improvement began 1 month after LSG. In the OGTT, lower increase in blood glucose after 1 and 2 h with lesser insulin concentrations needed to recover glucose homeostasis was observed during follow-ups. Overtime average glucose concentrations in CGM significantly decreased during follow-ups. Lipid metabolism improved in terms of HDL and triglycerides.
